# Astaxanthin attenuates cigarette smoke-induced small airway remodeling via the AKT1 signaling pathway

**DOI:** 10.1186/s12931-024-02768-4

**Published:** 2024-03-30

**Authors:** Haidong Ding, Liming Yan, Yu Wang, Ye Lu, Mingming Deng, Yingxi Wang, Qiuyue Wang, Xiaoming Zhou

**Affiliations:** 1 Department of Pulmonary and Critical Care Medicine, Affiliated Hospital of Inner Mongolia University for the Nationalities, Tongliao, China; 2 Jiangsu Provincial Key Laboratory of Geriatrics, Department of Geriatrics, The First Affiliated Hospital, Nanjing Medical University, Nanjing, China; 3 Department of Pulmonary and Critical Care Medicine, The Second Hospital of Dalian Medical University, Dalian, China; 4 Department of Pulmonary and Critical Care Medicine, Shengjing Hospital of China Medical University, Shenyang, 110004 Liaoning Province China; 5 Department of Pulmonary and Critical Care Medicine, Center of Respiratory Medicine, National Clinical Research Center for Respiratory Diseases, China-Japan Friendship Hospital, Beijing, China; 6 Department of Pulmonary and Critical Care Medicine, First Hospital of China Medical University, Shenyang, China; 7 Respiratory Department, Center for Pulmonary Vascular Diseases, Fuwai Hospital, National Center for Cardiovascular Diseases, Chinese Academy of Medical Sciences and Peking Union Medical College, Beijing, China

**Keywords:** Chronic obstructive pulmonary disease, Small airway remodeling

## Abstract

**Background:**

Astaxanthin (AXT) is a keto-carotenoid with a variety of biological functions, including antioxidant and antifibrotic effects. Small airway remodeling is the main pathology of chronic obstructive pulmonary disease (COPD) and is caused by epithelial-to-mesenchymal transition (EMT) and fibroblast differentiation and proliferation. Effective therapies are still lacking. This study aimed to investigate the role of AXT in small airway remodeling in COPD and its underlying mechanisms.

**Methods:**

First, the model of COPD mice was established by cigarette smoke (CS) exposure combined with intraperitoneal injection of cigarette smoke extract (CSE). The effects of AXT on the morphology of CS combined with CSE -induced emphysema, EMT, and small airway remodeling by using Hematoxylin-eosin (H&E) staining, immunohistochemical staining, and western blot. In addition, *in vitro experiments*, the effects of AXT on CSE induced-EMT and fibroblast function were further explored. Next, to explore the specific mechanisms underlying the protective effects of AXT in COPD, potential targets of AXT in COPD were analyzed using network pharmacology. Finally, the possible mechanism was verified through molecular docking and in vitro experiments.

**Results:**

AXT alleviated pulmonary emphysema, EMT, and small airway remodeling in a CS combined with CSE -induced mouse model. In addition, AXT inhibited the EMT process in airway cells and the differentiation and proliferation of fibroblasts. Mechanistically, AXT inhibited myofibroblast activation by directly binding to and suppressing the phosphorylation of AKT1. Therefore, our results show that AXT protects against small airway remodeling by inhibiting AKT1.

**Conclusions:**

The present study identified and illustrated a new food function of AXT, indicating that AXT could be used in the therapy of COPD-induced small airway remodeling.

**Supplementary Information:**

The online version contains supplementary material available at 10.1186/s12931-024-02768-4.

## Background

Chronic obstructive pulmonary disease (COPD) is a progressive lung disease that represents a significant global burden [1–3], with smoking and ambient particulate matter being the main risk factors [2, 4, 5]. Persistent airway inflammation is the main pathology of COPD, which leads to airway remodeling and small airway obstruction [6], presenting as an increase in the overall thickness of the airway wall [7]. This increase in wall thickness stems from epithelial changes, mucoid plugs, increased density of inflammatory cells, smooth muscle hyperplasia, and fibrosis [8], leading to an irreversible loss of lung function [9]. Current pharmacological treatments are directed toward reducing airway inflammation, boosting the endogenous levels of antioxidants, and relieving airway contraction and sputum production but do not suppress the progression of the disease due to irreversible small airway remodeling [10]. Therefore, exploring therapies targeting small airway remodeling in COPD may be beneficial for COPD management.

Astaxanthin (AXT) is a lipid-soluble keto carotenoid. Due to its powerful anti-inflammatory and antioxidant effects, AXT has attracted growing interest as a multi-target drug for various diseases [11]. A systematic review and meta-analysis revealed that astaxanthin showed antioxidant effects after a 3-week intervention, particularly at high dosages [12]. Two previous studies reported that AXT could decrease the number of inflammatory cells in bronchoalveolar lavage fluid (BALF), suggesting the anti-inflammatory effects of AXT [13, 14]. AXT scavenges glucose-induced reactive oxygen species from the mitochondria, thus suppressing peritoneal fibrosis via its antioxidative and anti-inflammatory activities. In addition, AXT shows novel therapeutic potential for pancreatic cancer [15] and colon cancer [16] by reversing the epithelial-mesenchymal transition (EMT) process. However, the effects of AXT on cigarette smoking-induced EMT and small airway remodeling remain unclear.

In this study, we hypothesized that astaxanthin might play a potential protective role against cigarette smoke-induced EMT in the small airway, thus inhibiting small airway remodeling. A cigarette smoke-exposed mouse model was used to evaluate the protective effects of AXT on cigarette smoke extract (CSE)-induced EMT and small airway remodeling. Network pharmacology and further experiments were conducted to explore the mechanisms of action of AXT.

## Methods

### Chemicals

AXT (purity ≥ 97%; CAS Number: 472-61-7) with a molecular weight of 596.8 was purchased from Sigma-Aldrich (St. Louis, MO, USA) and was used for in vitro experiments. The AXT powder (food grade, 10%, w/w) used for the animal model was purchased from Fengzu Biotech (Xi’an, China).

### Preparation of CSE

Marlboro cigarettes (Philip Morris Companies, 0.8 mg nicotine, 10 mg tar and 10 mg carbon monoxide per cigarette) were using to prepare CSE, as previously described [17]. Freshly prepared CSE was used for each experiment.

### Animals and treatments

Sixty male C57BL/6J mice (8–10 weeks) were purchased from Liaoning Changsheng Biotechnology Company (Benxi, China) and were housed under specific pathogen-free conditions. Mice were randomly divided into five groups (*n* = 12 per group): (1) control group, (2) CSE group (cigarette smoke [CS]exposure + CSE injected intraperitoneally group), (3) CSE group + low dose (10 mg/kg) of AXT (CS exposure combined CSE intraperitoneal injection + L-AXT), (4) CSE group + medium dose (50 mg/kg) of AXT (CS exposure combined CSE intraperitoneal injection + M-AXT), and (5) CSE group + high dose (100 mg/kg) of AXT (CS exposure combined CSE intraperitoneal injection + H-AXT). Mice in the control group were maintained in fresh air and injected intraperitoneally with 300 µL of vehicle (phosphate-buffered saline [PBS]) on days 1, 12, and 23. Briefly, mice from model group and three AXT group were exposed to CS in a sealed box with ventilation holes for 1 month (1 h twice per day, 6 days per week), and mice were intraperitoneally injected with 300 µL of CSE-PBS solution on days 1, 12, and 23. CS exposure was not performed on the day of CSE intraperitoneal injection.

In the three AXT groups, AXT dissolved in 100 µL of olive oil (vehicle) was astaxanthin was administered by oral gavage 30 min after per CS exposure and two hours following the intraperitoneal injection of CSE at day 1, 12, and 23. The control and CSE group were given 0.1 ml of PBS at the scheduled time points. The mice were sacrificed on the 29th day, 24 h after the last exposure to CS. In the preliminary experiment, mice were randomly divided into four groups (*n* = 5 per group): (1) control group, (2) CSE group (CS exposure combined CSE intraperitoneal injection ), (3) CS exposure combined CSE intraperitoneal injection + high dose (100 mg/kg) of AXT (CSE + H-AXT), 4) CSE + vehicle group (CS exposure combined CSE intraperitoneal injection + olive oil). In vehicle group, 100 µL olive oil was administered via oral gavage. The weight of each group was recorded, and compared with the CSE group, there was no significant weight change in the vehicle group, indicating that olive oil, as a carrier, had no effect on body weight (Supplementary Fig. 1). This study was approved by the Ethics Committee of China Medical University (No. KT2018061).

### Tissue collection and lung histopathology

After the mice were sacrificed, the lung tissue was rapidly collected and fixed with 4% paraformaldehyde, followed by gradient dehydration in alcohol and paraffin embedding. After the sample paraffin block was cut into 5 μm sections, hematoxylin and eosin (H&E) staining or immunohistochemical staining was performed. Histopathological sections of the lungs were observed under a light microscope and photographed.

### Histology of alveolar enlargement and airway remodeling

Paraffin sections were stained with H&E and Masson’s trichrome to examine histological changes. The morphology of the lungs harvested from different groups was assessed for emphysema changes based on the mean linear intercept (MLI) and mean alveolar number (MAN), as previously described (×100 magnification) [17]. Longitudinal sections were stained with Masson’s trichrome. Bronchioles with an internal diameter of 150–200 μm were selected in a blinded manner and observed and photographed [18]. The area of collagen deposition (µm^2^) was assessed in a minimum of four small airways (perimeter of bronchial basement membrane [Pbm] < 1000 μm) per section. There was no statistically significant difference in Pbm between groups. The area of trichrome staining was measured, normalized to Pbm (µm), and quantified using ImageJ software (Version 1.49 h, NIH, New York City, USA) [18–20]. The number of cells (nuclei) in the epithelium was assessed by H&E staining in a minimum of four small airways (Pbm < 1,000 μm) per section, normalized to Pbm (µm), and quantified using ImageJ software [20].

### Cell culture and treatment

16HBE (human bronchial epithelial cells) and MRC-5(human fetal lung fibroblast cells) were obtained from the China Infrastructure of Cell Line Resources. 16HBE cells were cultured in RPMI 1640 medium supplemented with 10% fetal bovine serum (FBS). MRC-5 cells were cultured in minimum essential medium (MEM) supplemented with 10% FBS. AXT was dissolved in dimethyl sulfoxide (DMSO) and diluted to a final concentration of DMSO < 0.1%. Cells were treated with AST at various concentrations (50 and 100 µM) or DMSO as a control and then stimulated with 2.5% CSE up to 48 h.

### Western blot

Western blotting was performed as previously described [21]. The following primary antibodies were used: anti-fibronectin (1:1000, Proteintech), anti-collagen I (1:1000, Proteintech), anti-α-SMA (1:1000, Cell Signaling Technology), anti-AKT1 (1:1000, Cell Signaling Technology), anti-phospho-AKT1 (Ser473) (1:1000, Cell Signaling Technology), and anti-β-actin (1:1000, Cell Signaling Technology).

### Cell viability

Cell viability was determined using a cell counting kit-8 (CCK8) assay following previously study [22]. MRC-5 cells were seeded into 96-well plates at a density of 1 × 10^4^ cells/well. The cells were treated with different concentrations of AXT with or without CSE. For the CCK8 assay, 10 µL CCK8 solution (C0038) was obtained from Beyotime Technology Corp., Ltd. Shanghai, China.

### Network pharmacology

The chemical composition information of AXT was collected from the PubChem database. Based on the searched structural formulae, potential targets of the composition were collected from the Swiss target and PharmMapper databases. Targets related to “Chronic obstructive pulmonary disease” were determined using data from the GeneCards and Online Mendelian Inheritance in Man (OMIM) databases.

The interaction target genes of compounds and diseases were introduced into the STRING database (http://sting-db.org) to obtain the target protein interaction network relationship. Cytoscape 3.7.2 software was used to import the protein-protein interaction (PPI) analysis to construct a PPI network; genes whose degree, betweenness centrality, and closeness centrality were greater than their respective medians were selected as core target genes. R software was used to import the PPI data as the number of connected nodes of the core gene and the histogram of the first 30 core genes. Kyoto Encyclopedia of Genes and Genomes (KEGG) pathway and Gene Ontology (GO) enrichment analyses were performed using Bioconductor.

### Molecular docking

The binding potential between AXT and the candidate targets from network pharmacology analysis was explored using molecular docking analysis of AXT. The AKT1 protein crystal structure was obtained from the UniProt database (UniProt ID: P31750; predicted structure using AlphaFold: AF-P31750-F1). The 3D structure of the small molecule AXT was obtained from the PubChem database (PubChem CID:5,281,224), and energy minimization was performed using AVOGADR 1.2.0 under the MMFF94 force field. AutoDock Vina 1.1.2 software was used for molecular docking analysis. Hydrogenation of all receptor proteins was performed using PyMol Academic Open-Source Edition. The hub gene proteins and major components were then converted to PDBQT format tools using 1.5.6 software by AutoDock. AutoDock Tools 1.5.6 was used to construct a crystal structure docking grid box for each target. The molecule with the lowest binding energy for each active compound in the docked conformation was then semi-flexibly docked by comparison with the original ligand and intermolecular interactions. The center coordinates and size of the box were determined to evaluate the interactions. Finally, the docking results were visualized using PyMOL software.

### Statistical analysis

The results are presented as the mean ± standard error of the mean (SEM). Statistical analysis was performed by one-way analysis of variance (ANOVA) followed by Tukey’s multiple-comparison test using GraphPad Prism software (CA, US). A two-sided *p* value of less than 0.05 was considered significantly different.

## Results

### AXT alleviated CSE-induced pulmonary emphysema and small airway remodeling in mice

**Fig. 1:**
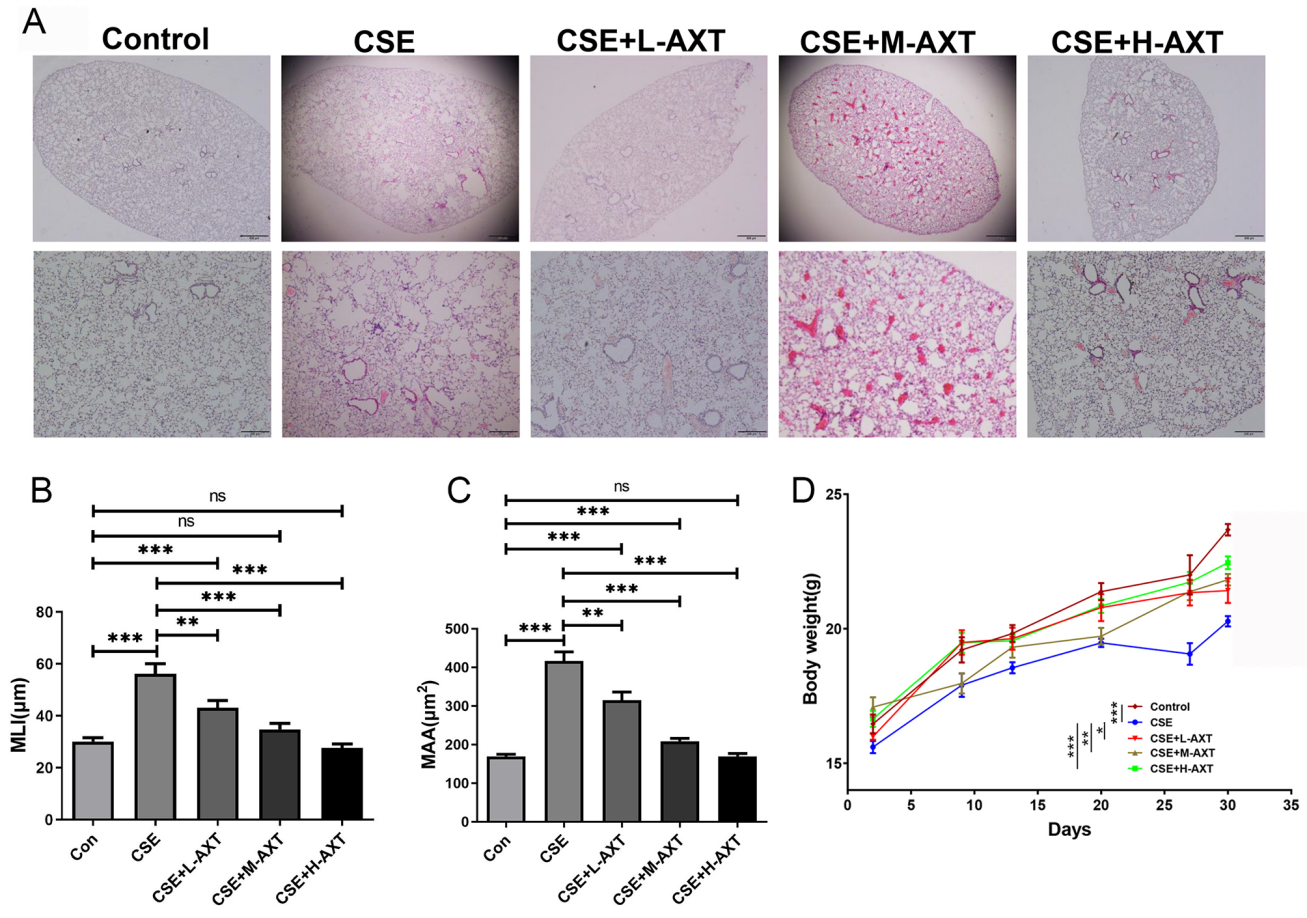
AXT alleviated CS combined with CSE -induced pulmonary emphysema (**A**): HE‑staining of lung tissue: (1) control group, (2) CS exposure combined CSE intraperitoneal injection (CSE) group, (3) CS exposure combined CSE intraperitoneal injection + 10 mg/kg body weight of AXT (CSE + L-AXT), (4) CS exposure combined CSE intraperitoneal injection + 50 mg/kg body weight of AXT (CSE + M-AXT), and (5) CS exposure combined CSE intraperitoneal injection + 100 mg/kg body weight of AXT (CSE + H-AXT); (**B**): Morphometric measurements of mean linear intercept (MLI) (µm); (**C**): Morphometric measurements of mean alveolar area (MAA) (µm^2^); (**D**): The weight of mice in each groups; *p < 0.05; **p < 0.01; *** *p* < 0.001

Emphysema is a cardinal feature of the pathogenesis of COPD. Thus, to study the potential effect of AXT on COPD, we first examined histological changes. CS exposure combined CSE intraperitoneal injection induced alveolar enlargement, parenchymal destruction, and alveolar wall rupture, while treatment with AXT decreased the histological changes of CS combined with CSE-induced emphysema (Fig. 1A**).** Further analysis showed that AXT notably reversed the increased mean linear intercept (MLI) and mean alveolar area (MAA) in CS combined with CSE -treated mice in a concentration-dependent manner (Fig. 1B and C**).** In addition, mice in the CS combined with CSE group had significantly lower body weights, whereas the different-dose AXT treatment significantly attenuated CS combined with CSE -induced body weight loss (Fig. 1D). These results suggest that AXT significantly reverses CS combined with CSE -induced emphysema.

**Fig. 2:**
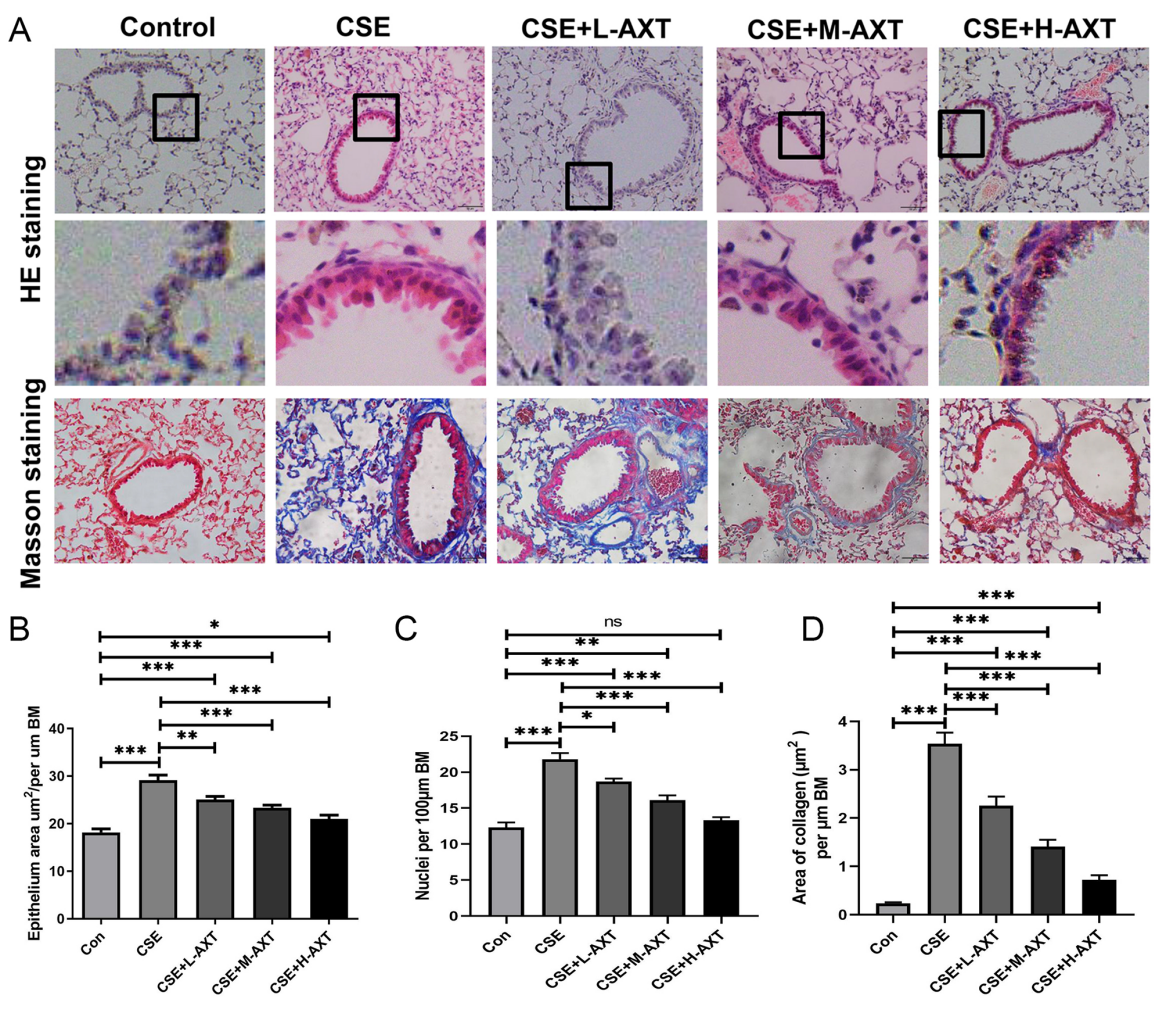
AXT alleviated CS combined with CSE -induced small airway fibrosis (**A**): HE‑staining, and Masson-staining of small airway: (1) control group, (2) cigarette smoke (CS) exposure combined cigarette smoke extract (CSE) intraperitoneal injection (CSE group), (3) CS exposure combined CSE intraperitoneal injection + 10 mg/kg body weight of AXT (CSE + L-AXT), (4) CS exposure combined CSE intraperitoneal injection + 50 mg/kg body weight of AXT (CSE + M-AXT), and (5) CS exposure combined CSE intraperitoneal injection + 100 mg/kg body weight of AXT (CSE + H-AXT); (**B**): The epithelium area by H&E staining was assessed in small airway and normalized to BM(bronchial basement membrane) (µm); (**C**): The cell (nuclei) number of the epithelium by H&E staining was assessed in small airway and normalized to BM (µm); (**D**): The peribronchiolar collagen deposition rate in each groups, normalized by per µm BM; *p < 0.05; **p < 0.01; *** *p* < 0.001

Small airway remodeling is a critical feature of COPD pathogenesis. Therefore, we examined the effects of AXT on CS combined with CSE -induced small airway remodeling. After one month of exposure to CS combined with CSE, the mice exhibited significant airway remodeling (Fig. 2A), showing an increased epithelial area in the small airway (Fig. 2B) and an increase in epithelial cell nuclei (Fig. 2C), indicating increased layers of epithelial cells in the small airway and collagen deposition in the epithelial wall (Fig. 2D). AXT treatment attenuated CS combined with CSE -induced small airway epithelial thickening and collagen deposition in a dose-dependent manner. These results indicate that AXT can alleviate CS combined with CSE -induced small airway fibrosis.

### AXT inhibited EMT in CSE-induced small airway remodeling both in mice and in vitro

**Fig. 3:**
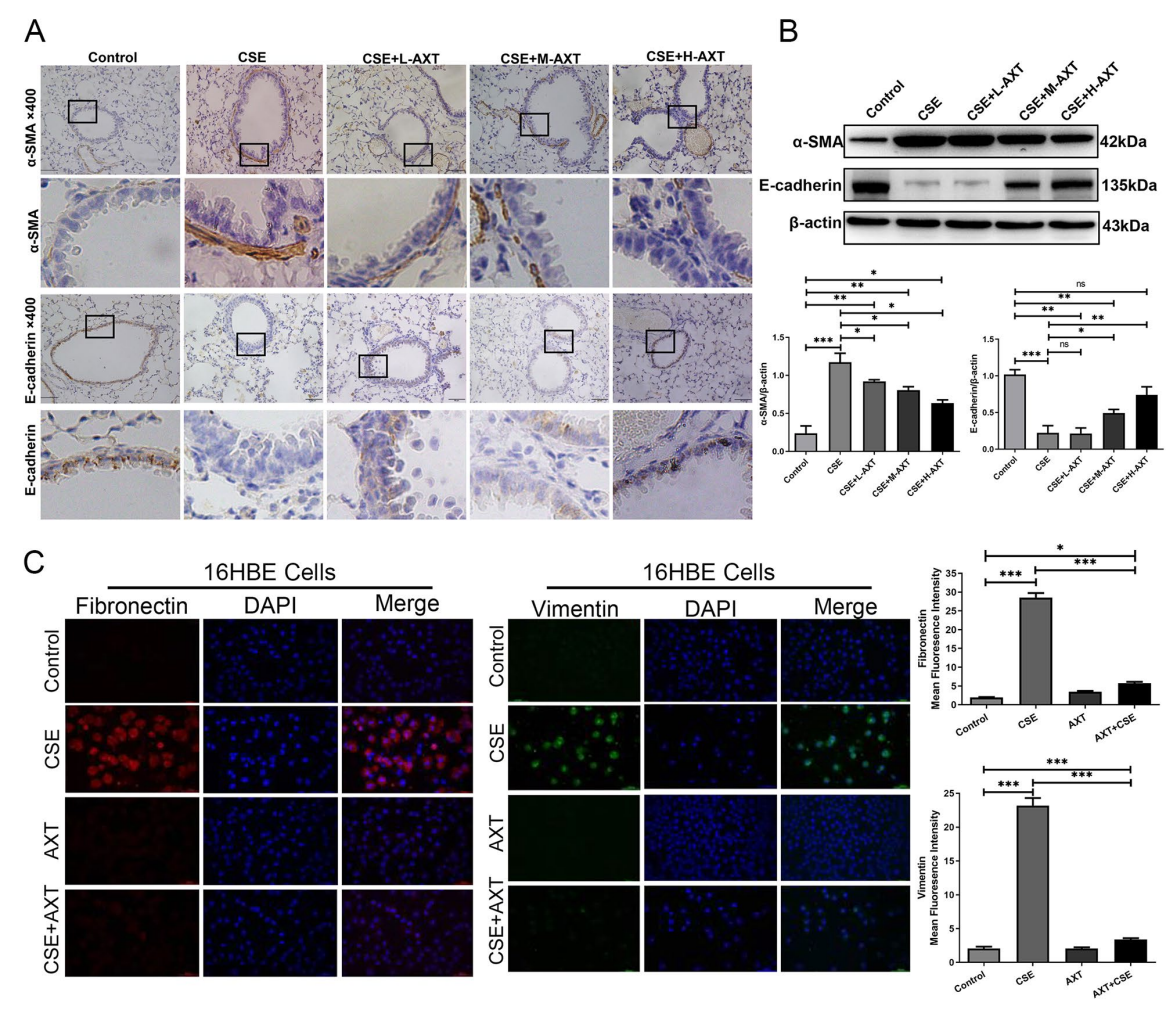
AXT inhibited EMT in CS combined with CSE -induced small airway remodeling in mice and in CSE -induced in small airway remodeling in vitro (**A**): IHC staining of α-SMA, and E-cadherin in mice small airway; (**B**): The protein level of α-SMA, and E-cadherin in mice lung tissue; (**C**): The expression of vimentin and fibronectin in 16HBE cells was shown by immunofluorescence staining. *p < 0.05; **p < 0.01; *** *p* < 0.001

EMT is a contributing factor to airway remodeling and is considered a critical mechanism in the pathogenesis of COPD [23, 24]. Therefore, we investigated the effect of AXT on EMT in airway epithelial cells. Using immunohistochemistry, we found that CS combined with CSE exposure decreased the expression of the epithelial marker E-cadherin, while that of the mesenchymal marker α-SMA was increased by CSE exposure (Fig. 3A). The administration of AXT reversed this change in accordance with the changes in small airway remodeling observed in the animal experiment. Western blot analysis showed changes similar to the decrease in E-cadherin and increase in vimentin, fibronectin, and α-SMA expression after treated with CS combined with CSE, whereas AXT attenuated the CS combined with CSE -induced EMT change (Fig. 3B). In addition, in the in vitro experiment, the expression of vimentin and fibronectin in 16HBE cells increased after CSE stimulation, while AXT reduced the increase in vimentin and fibronectin expression, as shown by immunofluorescence staining (Fig. 3C).

### AXT attenuated CSE-induced fibroblast differentiation and proliferation

**Fig. 4:**
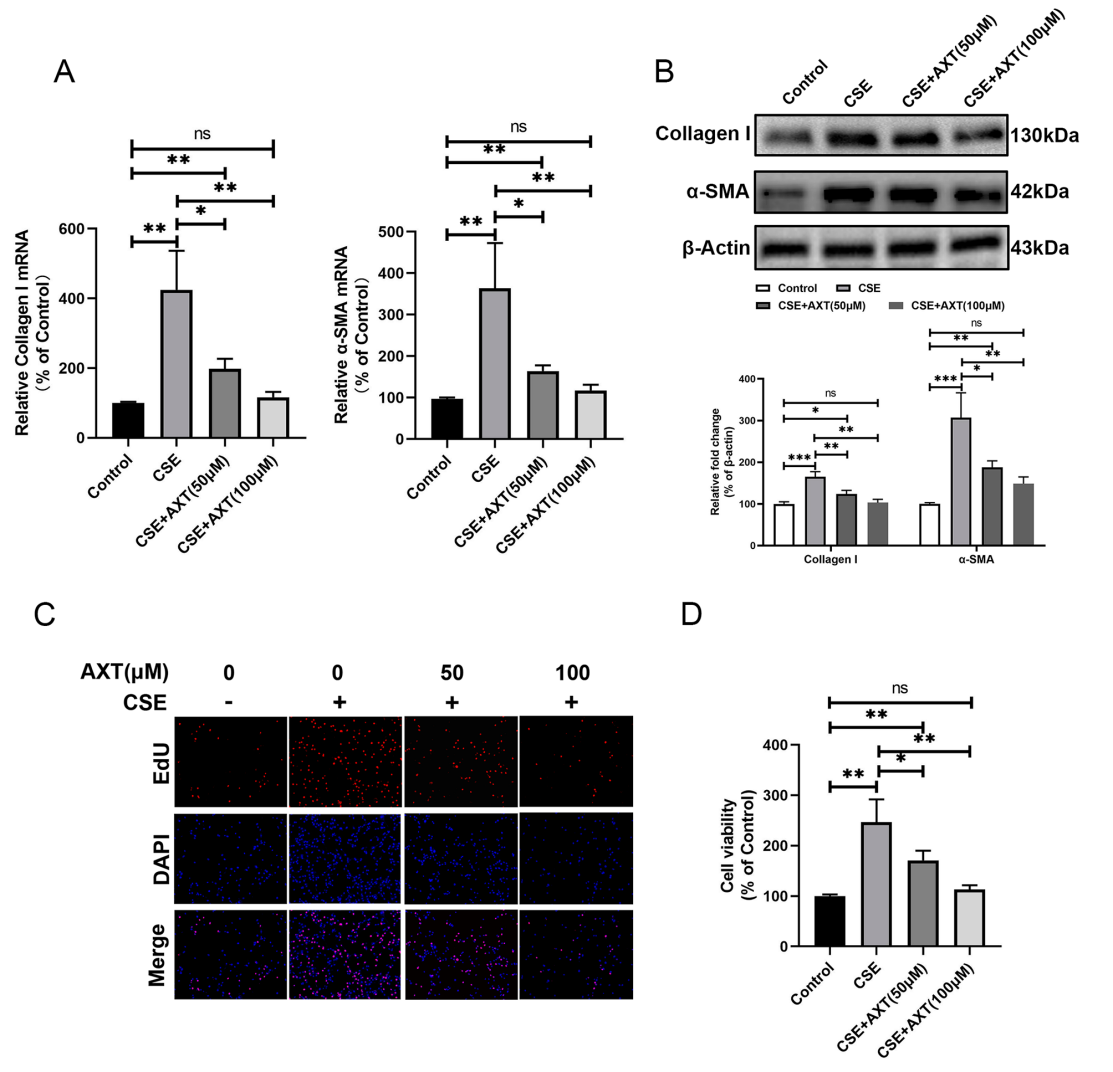
AXT attenuated CSE **-induced fibroblast differentiation and proliferation** The mRNA (**A**) and protein (**B**) expression levels of the classical EMT activation markers type I collagen and α-SMA were significantly upregulated in MRC5 cells after treatment with cigarette smoke extract (CSE), and AXT dose-dependently reduced CSE-induced upregulation of type I collagen and α-SMA levels; EdU staining (**C**) and CCK8 assay (**D**) were used to detect the effect of AXT on the proliferation of MRC-5 cells. *p < 0.05; **p < 0.01; ***p < 0.001

CS-induced fibroblast-to-myofibroblast transition is believed to contribute to airway remodeling in COPD. Therefore, we explored whether AXT affects fibroblast function. The results showed that the mRNA and protein expression levels of classical EMT activation markers type I collagen and α-SMA were significantly upregulated in MRC5 cells after treatment with CSE. Further results showed that AXT significantly reversed the CSE-induced upregulation of protein and mRNA levels of type I collagen and α-SMA in a dose-dependent manner (Fig. 4A and B).

In addition, we detected the effect of AXT on the proliferation of MRC-5 cells using EdU staining. As shown in Fig. 4C, proliferating cells with positive EdU staining significantly increased after treatment with CSE. Moreover, the combination of AXT and CSE significantly reversed the CSE-induced enhanced proliferative capacity. The CCK8 assay confirmed this result (Fig. 4D).

These results show that AXT significantly suppressed the proliferation of fibroblasts induced by CSE.

### Network pharmacology predicts the potential mechanism for AXT treatment of COPD

**Fig. 5:**
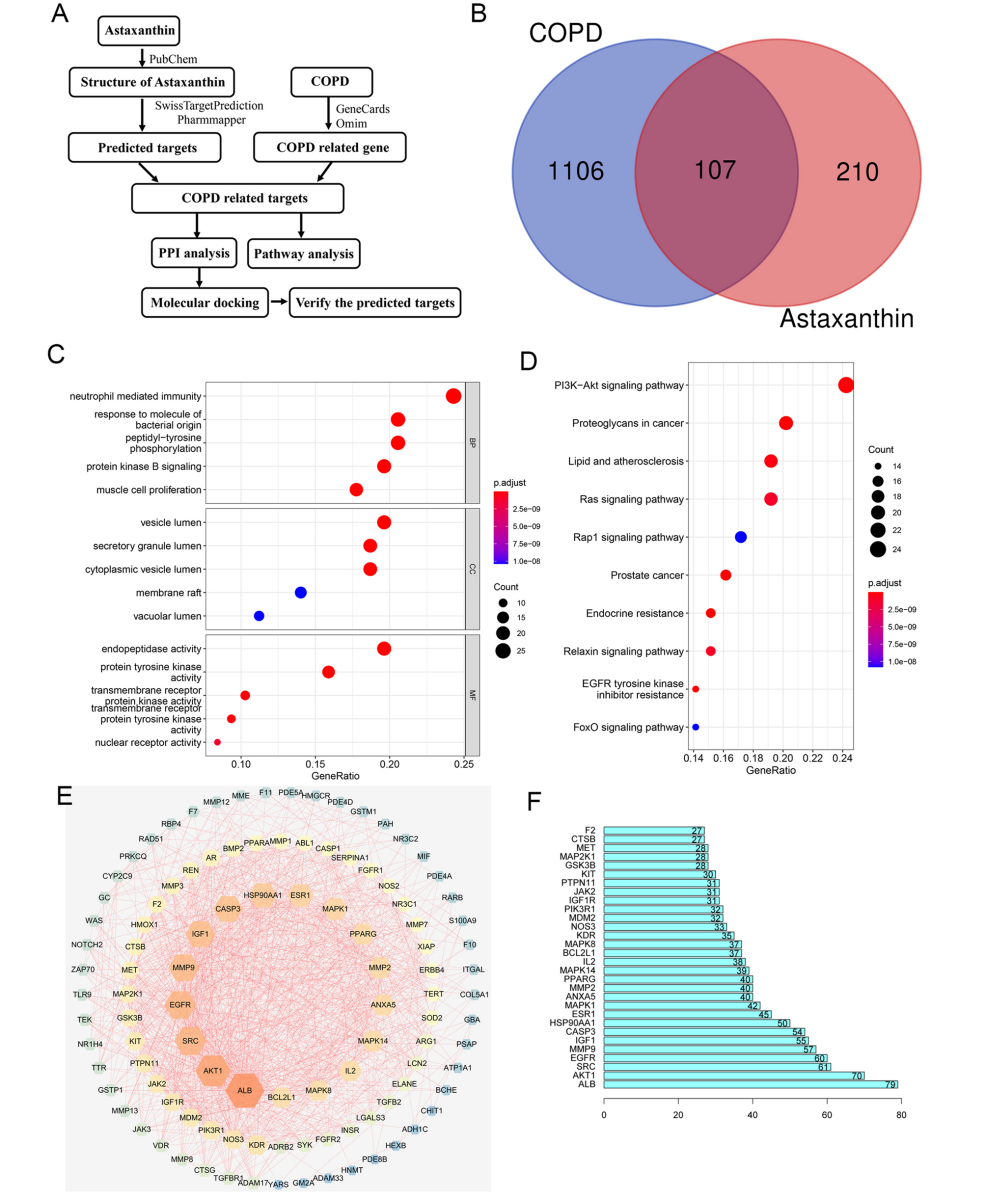
Network pharmacology predicts that the potential mechanism for AXT treatment of COPD (**A**): Process of target screening; (**B**): Venn diagrams of COPD-related gene and predicted targets of AXT; (**C**): GO enrichment of COPD-related targets; (**D**): KEGG enrichment of COPD-related targets; (**E**): Protein–protein interaction of COPD-related targets; (**F**): Ranking of core targets based on the protein–protein interaction network

To explore the specific mechanisms underlying the protective effects of AXT in COPD, potential targets of AXT in COPD were analyzed using network pharmacology (Fig. 5A**).** We collected 1106 potential target proteins of COPD and 210 target proteins of AXT from the database. In total, 107 intersecting targets were identified between AXT and COPD (Fig. 5B). After screening at *p* < 0.05, the top 10 pathways from GO (Fig. 5C) and the top 10 pathways from KEGG (Fig. 5D) were chosen according to risk factors. PPI network analysis was performed to analyze the interaction of 107 intersecting targets and 30 core target genes of AXT for COPD (Fig. 5E-F). Based on KEGG pathway enrichment analysis, 24 genes were enriched in the PI3K-AKT pathway. This suggests that the PI3K-AKT pathway is closely associated with the action of AXT in COPD.

### AXT directly binds and inhibits the phosphorylation level of AKT1

**Fig. 6:**
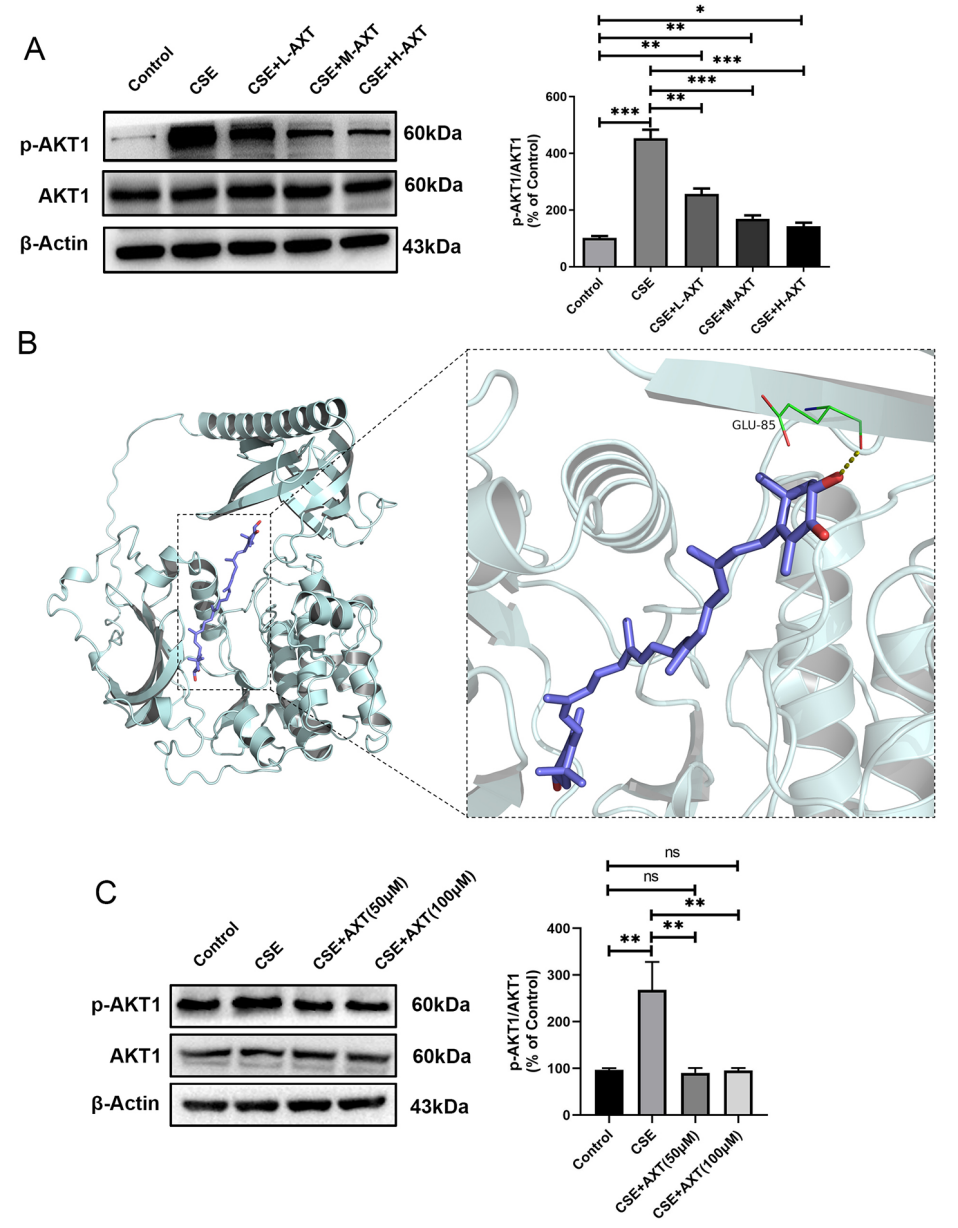
AXT directly binds and inhibits AKT1 (**A**): The protein level of AKT1 and phospho-AKT1 in mice lung tissue; (**B**): Interactive sites between AXT and AKT1 by docking analysis; (**C**): AXT increased phosphor-AKT1 expression dose-dependently in cigarette smoke extract(CSE)‐treated MRC5 cells; *p < 0.05; **p < 0.01; ***p < 0.001

Changes in the PI3K-AKT pathway were further investigated based on the results of network pharmacology analysis. AKT1 was chosen for the next analysis because it is the core molecule of the PI3K/AKT pathway and was ranked second in the PPI analysis. In animal experiments, decreased phospho-AKT1 levels were observed in the CSE group. The administration of AXT dramatically restored this reduction. Total AKT1 was used as the internal control (Fig. 6A). Next, molecular docking analysis was performed to verify whether AXT directly binds to AKT1. Nine different conformations of the AXT-AKT1 complex were generated using AutoDock software (Fig. 6B). After ranking the nine conformations according to Gibbs free energy, the first ranking was considered the best docking result, with a binding energy of -9.2 kcal/mol. AXT binds to the pocket of AKT1 protein surrounded by amino acids ILE84, CYS310, GLU191, LYS179, LYS158, VAL164, GLY157, ASP292, GLY294, PHE161, LEU295, TYR18, GLU17, and GLU85. Hydrogen bonds are formed between MY and GLU85. We further examined the expression of phospho-AKT1 and AKT1 after CSE stimulation of MRC5 cells. The level of phospho-AKT1 in MRC5 cells was significantly reduced after treatment with CSE and was restored to near baseline by AXT (Fig. 6C). These results suggest that AKT1 may be involved in the protective effects of AXT in CS combined with CSE -induced COPD models.

### AXT inhibited myofibroblast activation, suppressing the PI3K-AKT pathway

To further investigate whether AKT1 is involved in the anti-myofibroblast activation effects of AXT supplementation, we used SC79 to activate AKT1 phosphorylation.

**Fig. 7:**
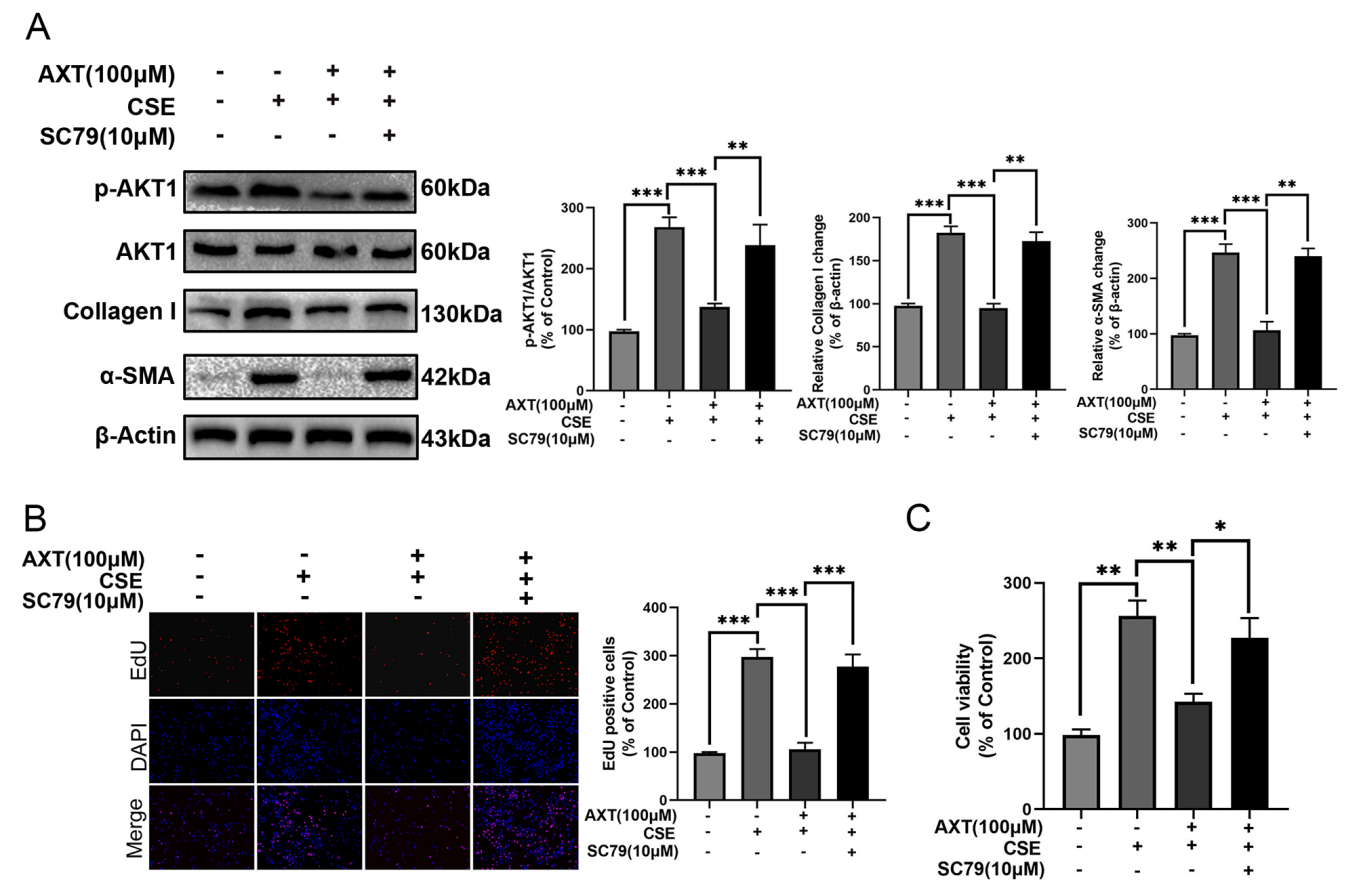
AXT inhibited myofibroblast activation and proliferation via inhibits AKT (**A**): Co-treatment of SC79 (Akt activitor, 10 µM) abolished the effect of AXT on expression of Collagen 1 and α-SMA; The alleviative effect of AXT on proliferation was reversed after application of SC79 in cigarette smoke extract(CSE)-treated MRC5 cells based on EdU (**B**) and CCK8 assays **(C);** *p < 0.05; **p < 0.01; ***p < 0.001

First, we explored the effect of AKT1 on the anti-myofibroblast-activating effect of AXT in CSE-stimulated MRC-5 cells. We found that AXT decreased the expression of type I collagen and α-SMA in CSE-treated MRC-5 cells and that these changes were abolished by SC79 treatment (Fig. 7A). In addition, the results of the EdU (Fig. 7B) and CCK8 assays (Fig. 7C) indicated that the upregulation of proliferative capacity was abolished in MRC-5 cells exposed to CSE after the combination of AXT and SC79. These results further suggest that AXT suppresses myofibroblast activation and proliferation via AKT1 signaling.

## Discussion

The small airways of patients with COPD are characterized by marked remodeling, with the overall thickness of the airway wall increased [8, 25]. Thus, preventing ongoing small airway remodeling is of great significance. A limited number of studies have shown that AXT attenuates CS-induced emphysema. However, the effect of AXT on the small airways remains unknown. Thus, in this study, we evaluated the effect of AXT on CS combined with CSE -induced small airway remodeling and found that, in addition to the attenuation of CS combined with CSE -induced emphysema, AXT can also restore small airway function by reducing small airway epithelial thickness and layers, as well as peribronchial collagen deposition. Overall, these results suggest that AXT can restore CS combined with CSE -induced small airway remodeling.

Airway remodeling is caused by abnormal tissue repair in response to injury, including CS and inflammation. Wound healing is tightly regulated by the interaction between the immune response to these stimuli and remodeling of the extracellular matrix by myofibroblasts, which are underneath epithelial cells undergoing EMT [26]. The extracellular matrix (ECM) is then remodeled by proteolytic enzymes, such as metalloproteinases, to re-establish the homeostatic balance of the tissue. Together, these changes lead to permanent alterations in the airway tissue architecture [8]. In this study, we found that the myofibroblast markers α-SMA and vimentin were increased in the airway epithelium, whereas E-cadherin was decreased after treated with CS combined with CSE, indicating epithelial cells lost epithelial proteins. Administration of AXT partially reversed EMT. Consistent with our results regarding EMT, a pulmonary fibrosis study showed that treatment of MRC-5 and A549 cells with AXT led to upregulation of E-cadherin and downregulation of vimentin in vitro [27]. The efficacy of AXT in promoting E-cadherin expression has also been validated in rats with bleomycin-induced lung fibrosis [27]. In addition, AXT attenuated CSE-induced fibroblast differentiation and proliferation.

To investigate the potential pathways involved in the mechanism of AXT in COPD, we performed network pharmacology analysis and found that the PI3K-AKT pathway was the most enriched pathway through multiple pathways and targets. Using western blot analysis, we confirmed the involvement of the PI3K-AKT pathway in the regulation of the small airway epithelium after CSE exposure. Molecular docking analysis indicated that AXT directly bound to AKT1. Further experimental results showed that AXT inhibited the phosphorylation level of AKT1 to protect mice against CS combined with CSE -induced airway remodeling. The PI3K/AKT pathway is interconnected with various inflammatory pathways in chronic inflammatory diseases, including those involved in tissue remodeling [28]. PI3K and AKT are key mediators that contribute to NF-κB activation induced by TNF-α [27, 29]. Taken together and combined with previous findings, these results suggest that AXT may play a protective role in CS combined with CSE -induced small airway remodeling and emphysema via the modulation of the PI3K/AKT pathway.

Although AXT plays a protective role in the progression of CS combined with CSE -induced small airway remodeling involving EMT, COPD is a heterogeneous disease that involves the regulation of multiple signaling networks. Thus, further studies should be performed to determine the specific and optimal usage of AXT.

## Conclusions

AXT protects against CS combined with CSE -induced airway remodeling by inhibiting EMT and myofibroblast activation, both in vivo and in vitro. Our study reveals novel molecular mechanisms underlying the protective role of AXT through the PI3K/AKT pathway. The present study identified and illustrated a new food function of AXT, indicating that AXT could be used in the therapy of COPD-induced small airway remodeling.

### Electronic supplementary material

Below is the link to the electronic supplementary material.

Supplementary Material 1: Supplementary Fig. 1 Weight change of mice in the preliminary experiment The weight of mice in control group, CS exposure combined with CSE intraperitoneal injection group (CSE group), CS exposure combined with CSE intraperitoneal injection + high dose (100 mg/kg) of AXT (CSE+H-AXT) group, and CSE + vehicle group (CS exposure combined with CSE intraperitoneal injection + olive oil) group.

Supplementary Material 2

## Data Availability

The data used to support the findings of this study are available from the corresponding author upon request.

## Abbreviations

COPD: chronic obstructive pulmonary disease
AXT: Astaxanthin; BALF, bronchoalveolar lavage fluid
EMT: epithelial-to-mesenchymal transition
CSE: cigarette smoke extract
CS: cigarette smoke
PBS: phosphate-buffered saline
MLI: mean linear intercept
MAN: mean alveolar number
FBS: fetal bovine serum
MEM: minimum essential medium
CCK8: cell counting kit-8
OMIM: Online Mendelian Inheritance in Man
PPI: protein-protein interaction
KEGG: Kyoto Encyclopedia of Genes and Genomes
GO: Gene Ontology
SEM: standard error of the mean
ANOVA: one-way analysis of variance
MAA: mean alveolar area
ECM: extracellular matrix
