# Supplementary material for: Astaxanthin attenuates cigarette smoke-induced small airway remodeling via the AKT1 signaling pathway
Source: Respir Res. 2024 Mar 30;25:148. doi: 10.1186/s12931-024-02768-4 (PMC10981815; doi:10.1186/s12931-024-02768-4)
Supplement: Supplementary file 1 — Supplementary Material 1: Supplementary Fig. 1 Weight change of mice in the preliminary experiment The weight of mice in control group, CS exposure combined with CSE intraperitoneal injection group (CSE group), CS exposure combined with CSE intraperitoneal injection + high dose (100 mg/kg) of AXT (CSE+H-AXT) group, and CSE + vehicle group (CS exposure combined with CSE intraperitoneal injection + olive oil) group. [file 12931_2024_2768_MOESM1_ESM.docx]

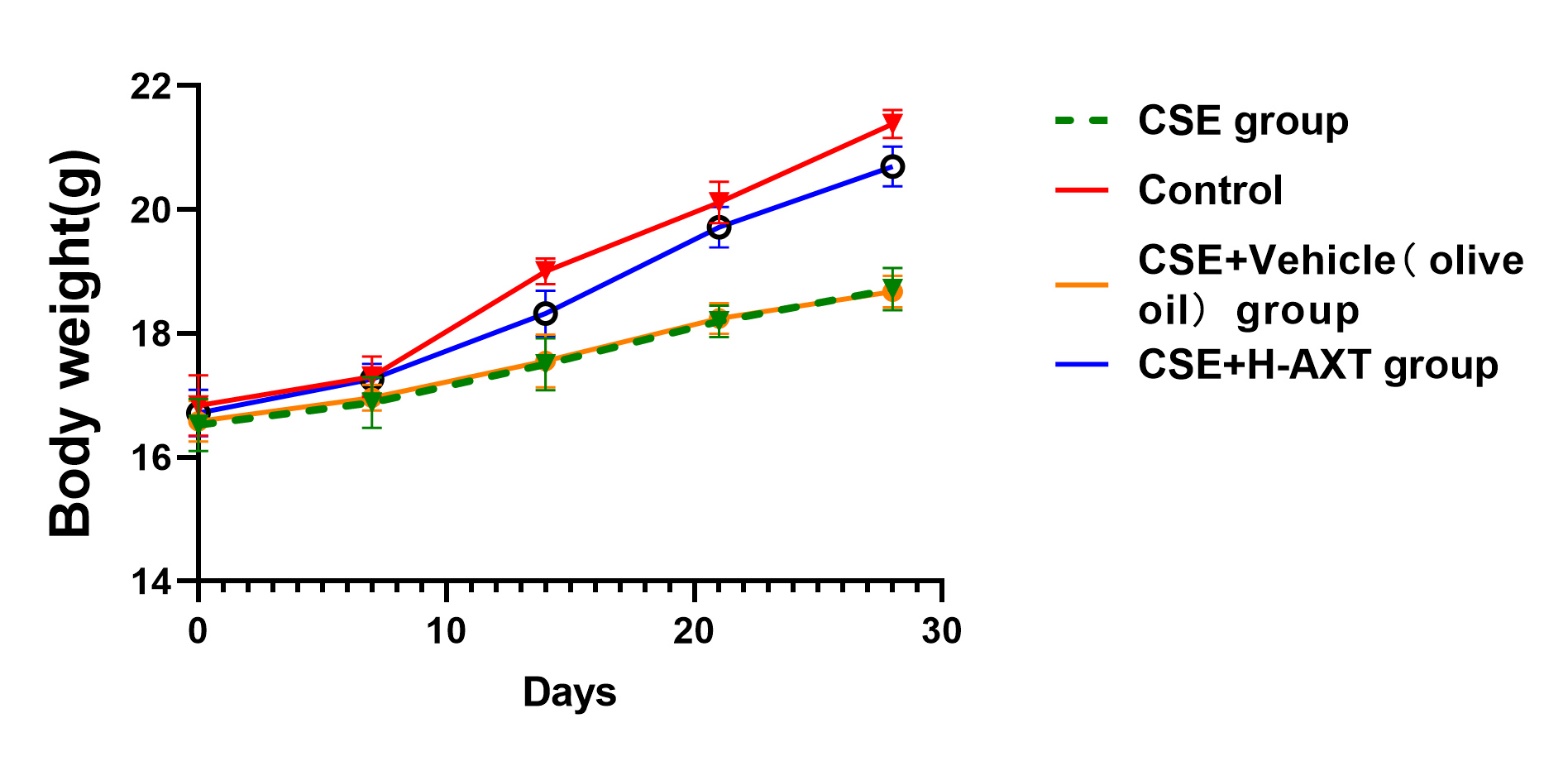


**Supplementary Figure.1 Weight change of mice in the preliminary experiment**

The weight of mice in control group, CS exposure combined with CSE intraperitoneal injection group (CSE group), CS exposure combined with CSE intraperitoneal injection + high dose (100 mg/kg) of AXT (CSE+H-AXT) group, and CSE + vehicle group (CS exposure combined with CSE intraperitoneal injection + olive oil) group.
