# Supplementary material for: Astaxanthin attenuates cigarette smoke-induced small airway remodeling via the AKT1 signaling pathway
Source: Respir Res. 2024 Mar 30;25:148. doi: 10.1186/s12931-024-02768-4 (PMC10981815; doi:10.1186/s12931-024-02768-4)

Fig.3B

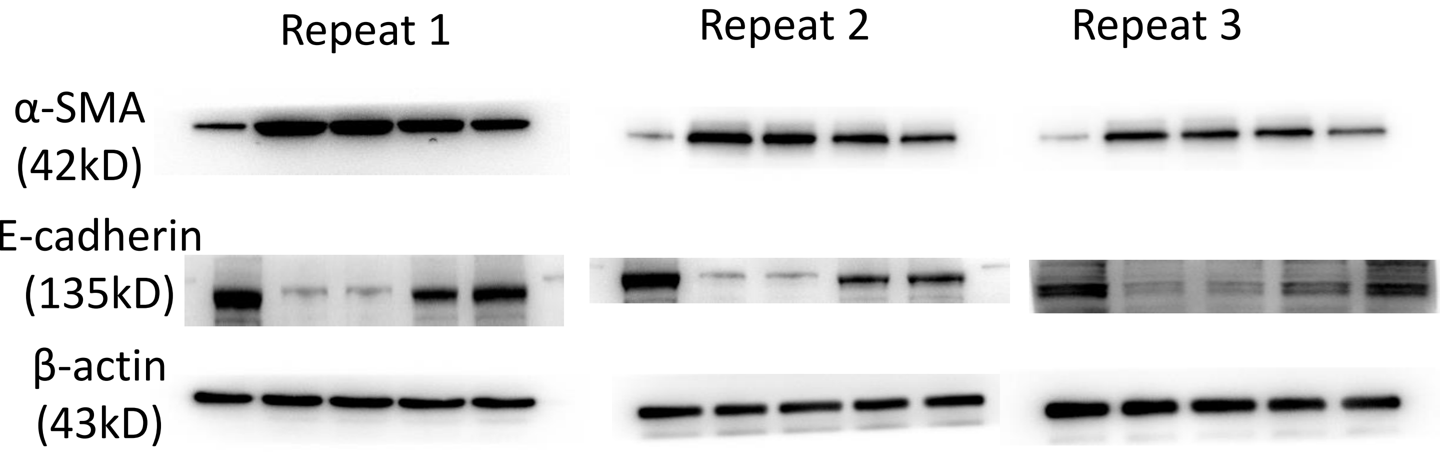
Repeat 1
Repeat 2
Repeat 3
α-SMA
(42kD)
E-cadherin
(135kD)
β-actin
(43kD)

Fig.4B

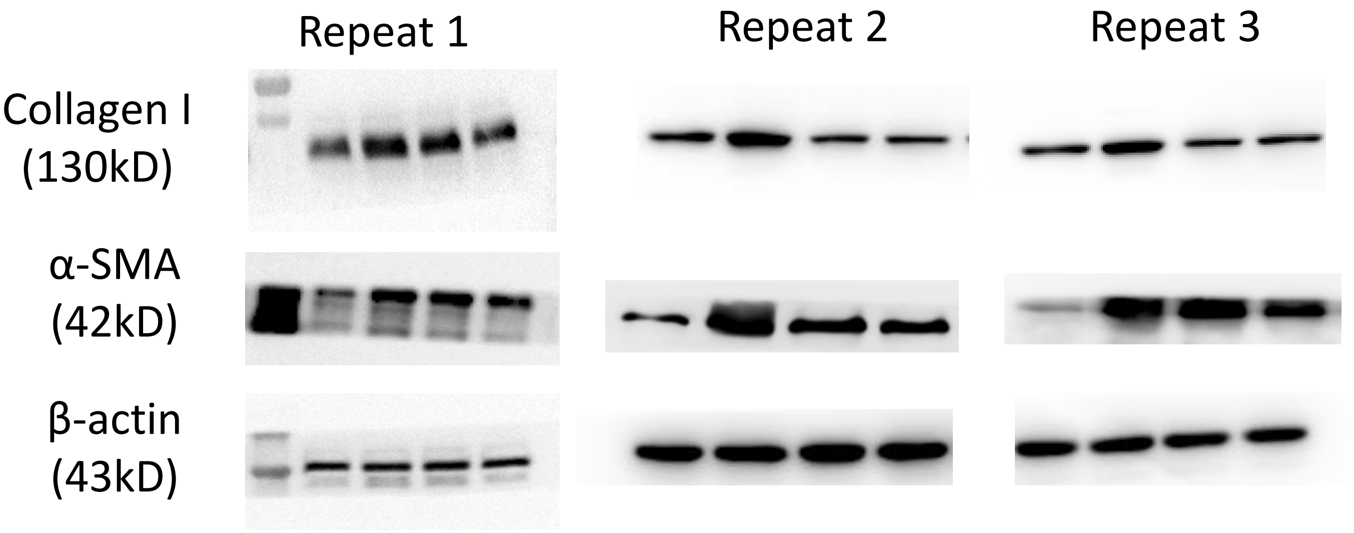
Repeat 1
Repeat 2
Repeat 3
Collagen I
(130kD)
α-SMA
(42kD)
β-actin
(43kD)

Fig.6A

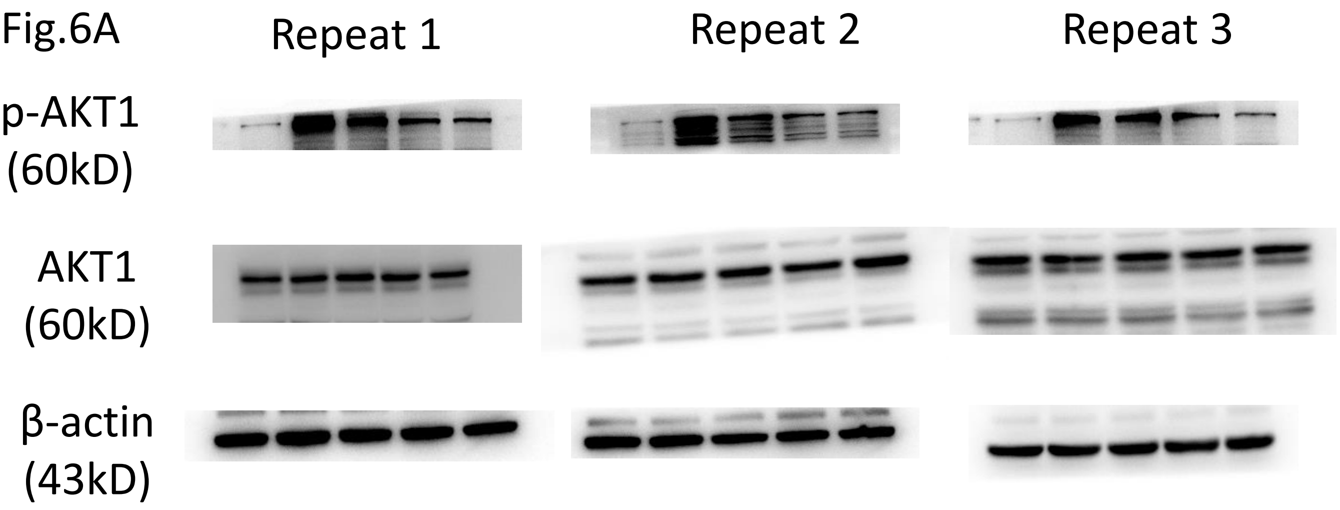

Fig.6C

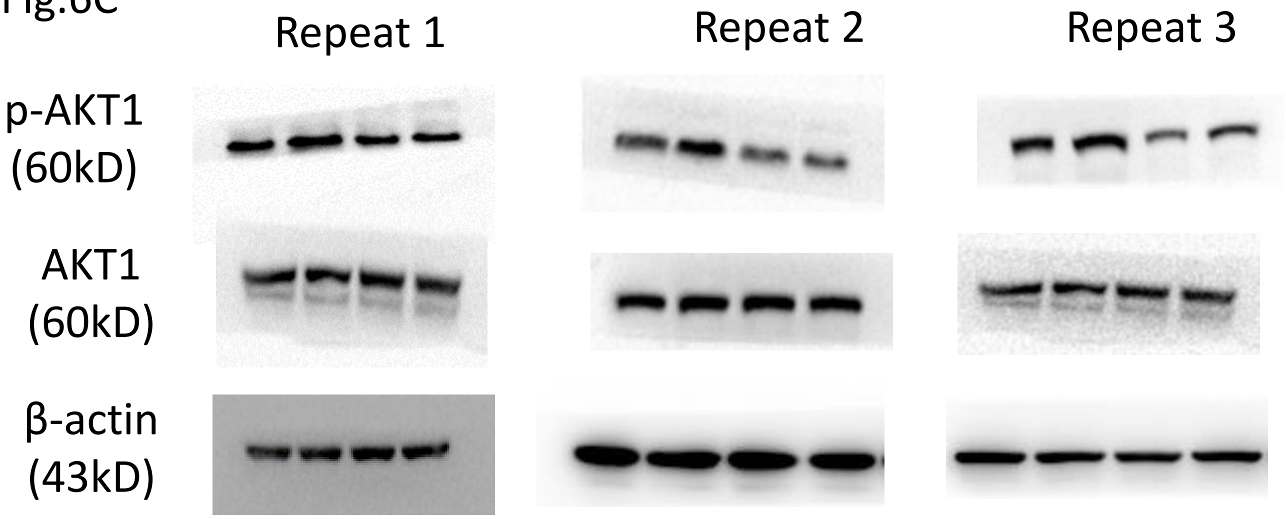

Fig.7A

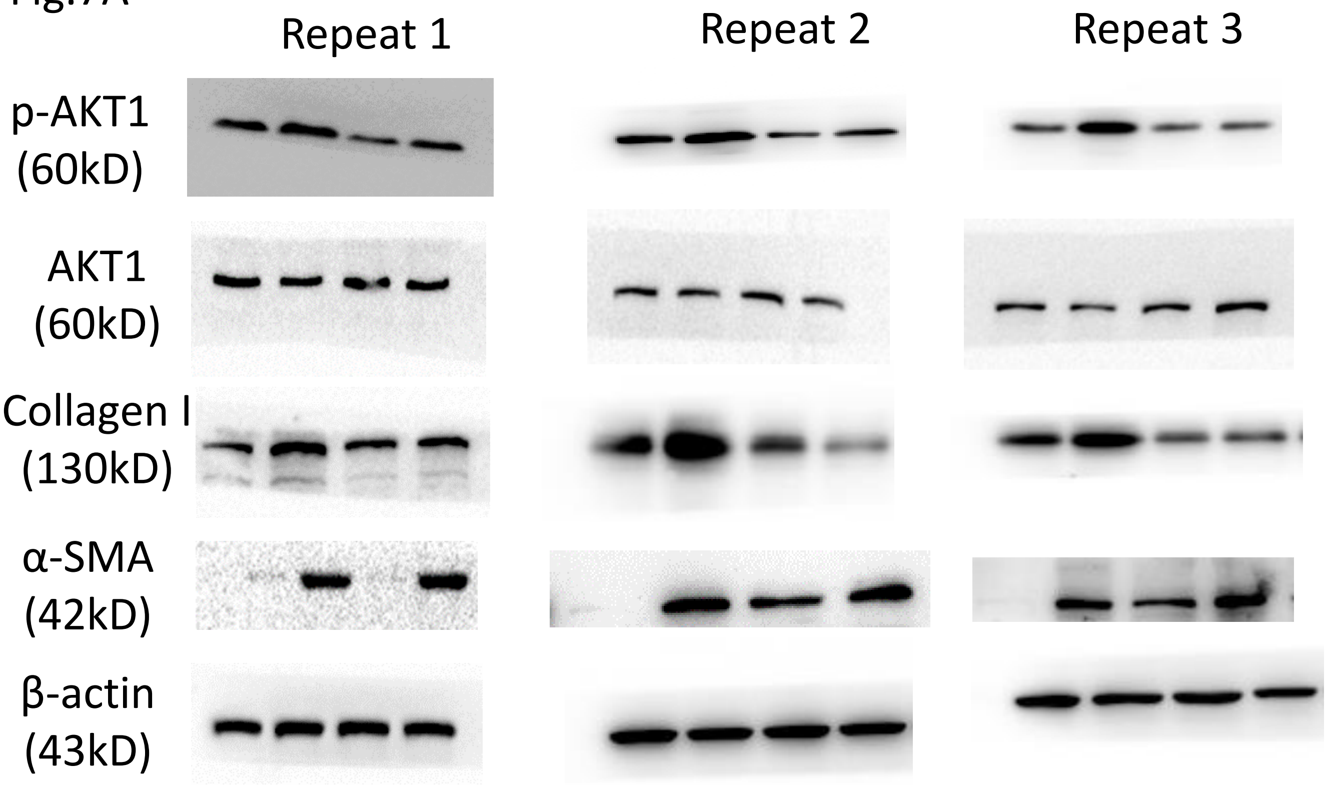

Supplement: Supplementary file 2 — Supplementary Material 2 [file 12931_2024_2768_MOESM2_ESM.pdf]
